# Distinct domains of the β1-subunit cytosolic N terminus control surface expression and functional properties of large-conductance calcium-activated potassium (BK) channels

**DOI:** 10.1074/jbc.M116.769505

**Published:** 2017-04-03

**Authors:** Lie Chen, Danlei Bi, Zen Huat Lu, Heather McClafferty, Michael J. Shipston

**Affiliations:** From the ‡ Centre for Integrative Physiology, College of Medicine and Veterinary Medicine, University of Edinburgh, Edinburgh EH8 9XD, Scotland, United Kingdom,; § PAPRSB Institute of Health Sciences, Universiti Brunei Darussalam, Jalan Tungku Link BE1410, Brunei Darussalam,; ¶ Neurodegenerative Disease Research Center, School of Life Sciences, University of Science and Technology of China, Hefei 230026, China,and; ‖ Division of Genetics and Genomics, The Roslin Institute, University of Edinburgh, Edinburgh EH25 9RG, Scotland, United Kingdom

**Keywords:** channel activation, electrophysiology, membrane trafficking, membrane transport, potassium channel

## Abstract

The properties and function of large-conductance calcium- and voltage-activated potassium (BK) channels are modified by the tissue-specific expression of regulatory β1-subunits. Although the short cytosolic N-terminal domain of the β1-subunit is important for controlling both BK channel trafficking and function, whether the same, or different, regions of the N terminus control these distinct processes remains unknown. Here we demonstrate that the first six N-terminal residues including Lys-3, Lys-4, and Leu-5 are critical for controlling functional regulation, but not trafficking, of BK channels. This membrane-distal region has features of an amphipathic helix that is predicted to control the orientation of the first transmembrane-spanning domain (TM1) of the β1-subunit. In contrast, a membrane-proximal leucine residue (Leu-17) controls trafficking without affecting functional coupling, an effect that is in part dependent on controlling efficient endoplasmic reticulum exit of the pore-forming α-subunit. Thus cell surface trafficking and functional coupling with BK channels are controlled by distinct domains of the β1-subunit N terminus.

## Introduction

Large-conductance calcium- and voltage-activated potassium channels are ubiquitously expressed and regulate a diverse array of physiological processes (1). This is associated with a wide functional diversity with the functional properties of BK^2^ channels being adapted to the physiological requirements of specific cell types. Disruption of BK channel function in human and animals is associated with a wide range of pathologies ranging from hypertension, autism, asthma, cancer, diabetes, and obesity to other disorders of the vascular, nervous, endocrine, and other systems.

The pore-forming α-subunit is encoded by a single gene (*KCNMA1*). However, BK channels in native tissues show distinct properties (1–3). This functional diversity is generated by multiple mechanisms including alternative splicing and post-translational modification of the α-subunit (3) as well as through assembly with a range of transmembrane accessory β- and γ-subunits (4, 5).

Regulatory β1-subunits, which have a distinct tissue distribution and play an important role in vascular and other smooth muscle, exert diverse functional effects on the α-subunit. Indeed, genetic deletion of β1-subunits in mice leads to hypertension, and mutations in the β1-subunit are associated with hypertension and asthma. β1-subunits consist of two transmembrane-spanning domains (TM1 and TM2) with short intracellular N- and C-terminal domains and a large extracellular loop coupling TM1 to TM2. β1-subunits control multiple aspects of BK channel function. The subunits control BK channel cell surface expression that is dependent on domains within both α- and β1-subunits (6–8). Importantly, β1-subunits also confer functional regulation of BK channel properties (6, 9, 10) including a large hyperpolarizing shift in the conductance-voltage relationship of macroscopic currents as a result of changes in apparent calcium sensitivity, a shift in magnesium sensitivity, and modification of gating through control of voltage sensing and slowing of channel activation and deactivation. Moreover, β1-subunits confer regulation by a diverse array of molecules including hormones, alcohol, fatty acids, and drugs (11).

Although β1-subunits confer such a diversity of modulation of BK channel function, specific domains and residues in the β1-subunit that are important for control are only beginning to emerge. For example, both the N and C termini of the β1-subunit have been implicated in the control of BK channel surface expression as well as the control of functional properties (7, 8, 10, 12–14). However, whether distinct regions of the β1-subunit confer different effects on channel trafficking compared with effects on functional coupling or sensitivity or similar regions control overlapping modes of regulation is poorly understood. Important in this regard is that studies, using both biochemical and biophysical/optical approaches, to investigate how β1-subunits and α-subunits assemble in the membrane are beginning to reveal the importance of the location of both the transmembrane and intracellular N termini of β1-subunits in relationship with transmembrane and other domains of the α-subunit (15, 16). Importantly, such studies suggest that the TM1 and TM2 of β1-subunits as well as the S0 domain of α-subunits have different trajectories through the plasma membrane.

Moreover, studies are beginning to reveal the role of specific amino acid residues in the short (18-amino-acid) N terminus of β1 in controlling both surface expression and functional coupling. For example, mutation of two basic residues (Lys-3 and Lys-4) largely abolishes gating charge movement in the absence of calcium, suggesting that these residues play a critical role in stabilizing the voltage sensor (10). In addition, mutant cycle analysis has suggested a role for a hydrophobic patch of residues (Leu-5, Val-6, and Met-7) and an electrostatic enhancing site (Glu-13 and Thr-14) that are proposed to mediate interactions of β1-subunits with different regions of the α-subunit to control magnesium and calcium sensitivity (12). Conversely, deletion of the entire N terminus abolishes functional coupling as well as the reported β1-subunit-induced enhancement of BK channel surface expression (7, 14), although residues important in the cytosolic N terminus of mammalian β1-subunits that control BK trafficking are not defined.

Increasing evidence also suggests that distinct regions of the β1-subunit N terminus may be targets for differential regulation. For example, amino acids in the membrane-proximal region of the N terminus are important for the ability of ω-3 fatty acids to activate the BK channel α·β1 complex (17, 18) and have been implicated in the potential beneficial effects of ω-3 fatty acids in blood pressure control. In contrast, somatic cancer mutations have been reported that cluster around the basic and hydrophobic residues in the more distal region of the N terminus (19, 20) with changes in BK channel activity linked to cancer cell proliferation and migration (21–23).

In this study, we sought to address whether the surface trafficking effects mediated by the β1-subunit N terminus required similar or distinct regions of the N terminus as those that control functional regulation. To address this issue, we exploited an integrated mutagenesis and deletion strategy coupled with surface expression and functional macroscopic current analysis. Here we demonstrate that distinct N-terminal residues of the β1-subunit control functional coupling to α-subunits and control α-subunit surface expression, respectively.

## Results

### Membrane-proximal domain of β1-subunit N terminus is required for enhanced trafficking of BK α-subunit ZERO variant

To address the role of the intracellular N terminus of the β1-subunit in enhancing the cell surface expression of the pore-forming α-subunit, we used a cell surface imaging assay to investigate the co-expression of β1-subunit deletion and site-directed mutants with a C-terminal myc (-myc) tag (β1-myc_c_) and the BK channel α-subunit ZERO variant with an extracellular N-terminal FLAG (FLAG-) tag.

**Figure 1.**
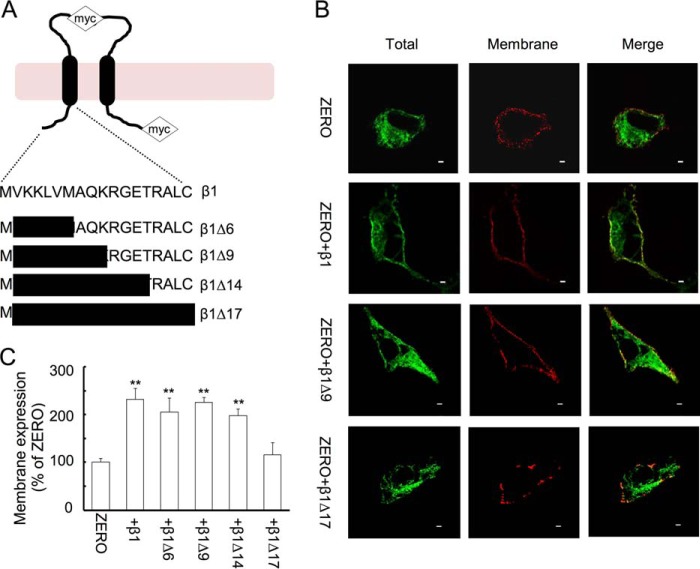
**Membrane-proximal residues of β1-subunit N terminus control surface expression and pore-forming α-subunit ZERO variants.** *A*, schematic of β1-subunit topology with residues of N-terminal deletion mutants indicated along with the C-terminal and extracellular myc tags used in these studies. *B*, representative single confocal images of the cell surface (membrane) and intracellular (permeabilized) expression of FLAG-ZERO determined in the presence and absence of wild-type β1 subunits with a C-terminal myc tag (β1-myc_c_) and respective mutants in HEK293 cells. *Scale bars*, 2 μm. *C*, quantification of cell surface membrane expression of FLAG-ZERO with the respective β1-subunit deletion mutants expressed as a percentage of FLAG-ZERO surface expression alone (100%). Data are means, and *error bars* represent S.E. (*N* > 8; *n* > 50/group). **, *p* < 0.01 compared with ZERO alone (ANOVA with Dunnett's post hoc test).

**Figure 2.**
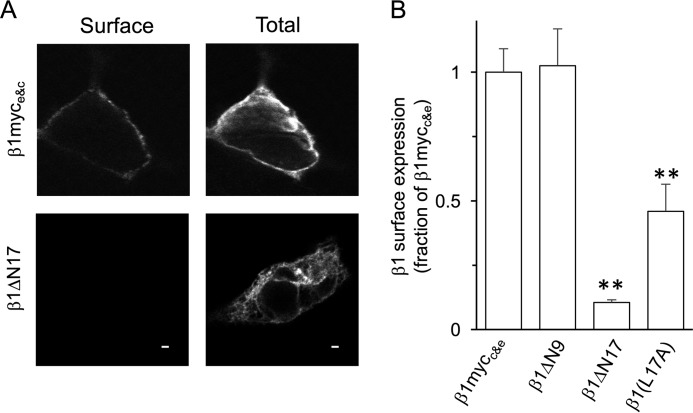
**N terminus of β1-subunit controls surface trafficking of β1-subunit alone.** *A*, representative single confocal images of the cell surface (membrane) and total (permeabilized) expression of wild-type and mutant β1-subunits with both C-terminal and extracellular myc tags (β1-myc_c&e_) in HEK293 cells. *Scale bars*, 2 μm. *B*, quantification of cell surface membrane expression of the respective β1-subunits expressed as a fraction of wild-type (β1-myc_c&e_) surface expression alone. Data are means, and *error bars* represent S.E. (*N* > 4; *n* > 30/group). **, *p* < 0.01 compared with β1-myc_c&e_ alone (ANOVA with Dunnett's post hoc test).

By analyzing the cell surface FLAG- signal levels under non-permeabilized conditions (expressed as a percentage of total α-subunit expression following membrane permeabilization) in anti-myc-positive HEK293 cells, we found that wild-type β1-subunits significantly enhanced the cell membrane surface expression of ZERO by more than 2-fold (232.3 ± 22.7%, *n* = 7; Fig. 1, *A–C*) in comparison with that of ZERO alone (100 ± 7.8%, *n* = 8). Deletion of the predicted N terminus of the β1-subunit (β1-Δ17-myc_c_ mutant with N-terminal amino acids 2–18 deleted) resulted in no significant enhancement of ZERO cell surface expression (Fig. 1, *A–C*). The loss of enhancement, however, was not the result of poor expression of ZERO. In fact, in transfected cells, the N terminus-deleted mutant β1-Δ17-myc_c_ displayed a similar robust expression level as that of wild-type β1-myc_c_, suggesting that changes in protein expression do not underlie the deficit in promoting surface expression of the ZERO α-subunit. Moreover, the physical interaction of β1-Δ17-myc_c_ and ZERO subunits was not disrupted as FLAG-ZERO and β1-Δ17-myc_c_ could co-immunoprecipitate in pulldown assays (data not shown). We asked whether the inability of the β1-Δ17-myc_c_ deletion mutant to promote cell surface expression of ZERO may be a consequence of the inability of the deletion mutant to traffic to the cell surface alone. To test this, we generated β1 mutants harboring an additional extracellular myc (myc_e_) tag to allow for the quantification of β1-subunit cell surface expression. Wild-type β1-myc_c&e_ mutants were efficiently trafficked to the cell surface alone. However, this was reduced to 10.3 ± 1.2% (*n* = 11) of wild-type levels with the N-terminal deletion mutant β1-Δ17-myc_c&e_ (Fig. 2, *A* and *B*). Thus, the N terminus of the β1-subunit is required for both efficient surface expression of the β1-subunit *per se* and the ability of the β1-subunit to promote ZERO α-subunit surface expression.

To define the domains of the intracellular N terminus important for the enhanced cell surface expression of ZERO subunits, we first took a deletion strategy to remove increasingly larger distal regions of the intracellular N terminus of the β1-subunit. Deletion of residues 1–6 or 2–10 (β1-Δ6-myc_c_ and β1-Δ9-myc_c_ constructs) of the β1-subunit N terminus promoted cell surface expression of ZERO by more than 2-fold and was not distinguishable from wild-type β1-subunit (Fig. 1, *B* and *C*). Deletion of residues 2–14 (β1-Δ14-myc_c_ construct) also significantly promoted cell surface expression by almost 1.8-fold, suggesting that the first 14 amino acids are largely dispensable for promoting cell surface expression of the ZERO subunit.

### Lysine 17 is important for β1-subunit enhancement of ZERO α-subunit surface expression

**Figure 3.**
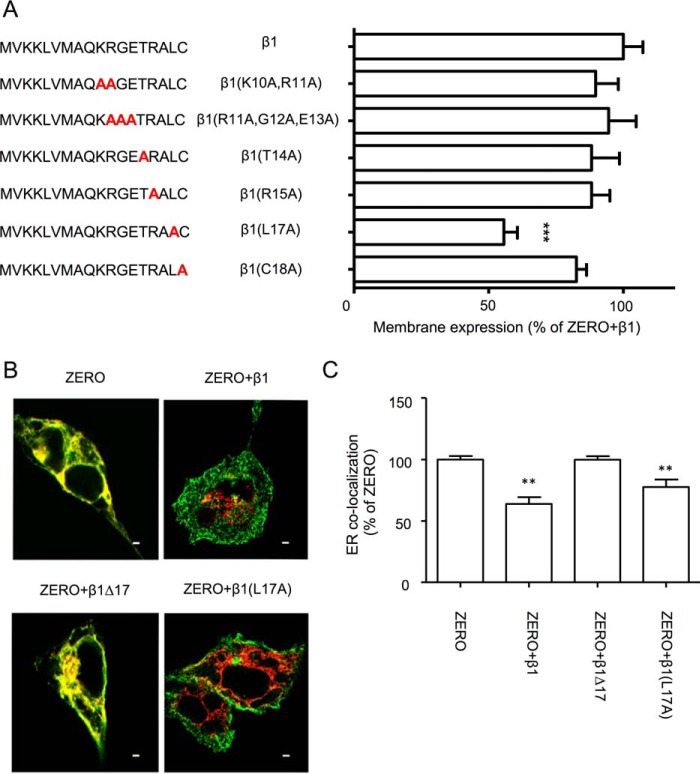
**Leu-17 of N-terminal β1-subunit controls surface expression BK α-subunit ZERO variant.** *A*, alanine substitution mutagenesis of membrane-proximal residues in the N terminus of -myc_c_-tagged β1-subunits and quantification of the respective cell surface FLAG-ZERO expression in the presence of each mutant. Data are normalized to FLAG-ZERO surface expression in the presence of wild-type β1-myc_c_ (100%). ***, *p* < 0.001. *B*, representative single confocal images of FLAG-ZERO (*green*) co-expressed with wild-type or mutant β1-subunits in HEK293 cells and the endoplasmic reticulum marker (pDsRed-ER) (*red*) with ER colocalization shown in *yellow. Scale bars*, 2 μm. *C*, bar chart of quantitative ER colocalization of ZERO (expressed as a percentage of the Pearson's correlation coefficient (*R* value) for ZERO alone (100% = *R* value of 0.82 ± 0.03) in the presence of the respective β1-L17A and β1-Δ17 mutants. Data are means, and *error bars* represent S.E. (*N* > 8; *n* > 50/group). **, *p* < 0.01 compared with ZERO alone (ANOVA with Dunnett's post hoc test).

To refine the N-terminal residues important for enhanced cell surface trafficking of ZERO promoted by the β1-subunit, we used an alanine substitution-scanning approach of residues immediately preceding residue 14 up to cysteine 18 at the membrane interface. We constructed single or multiple alanine-substituted mutations of C-terminal myc (myc_c_)-tagged β1-subunits β1-(K10A,R11Α)-myc_c_, β1-(R11Α,G12Α,Ε13Α)-myc_c_, β1-Τ14Α-myc_c_, β1-R15Α-myc_c_, β1-L17Α-myc_c_, and β1-C18A-myc_c_ and co-expressed each of them with FLAG-ZERO in HEK293 cells. The alanine-mutated β1-subunits promoted cell surface expression of ZERO subunits to a similar extent as wild-type β1-subunits except for β1-L17Α-myc_c_ (55.7 ± 5.0%, *n* = 9), which significantly decreased the enhanced membrane expression level of ZERO (Fig. 3*A*). This result suggests that Leu-17 of β1-subunits plays an important role in controlling the enhanced membrane trafficking of ZERO.

To understand how Leu-17 may control the β1-subunit enhancement of ZERO surface expression, we asked whether the β1-subunit β1-Δ17-myc_c_ is important for controlling endoplasmic reticulum (ER) exit of ZERO. We co-expressed FLAG-ZERO and wild-type β1-myc_c_ or β1 mutants together with pDsRed-ER, a vector that encodes a fusion protein. After deconvolution of confocal images, ZERO colocalization with the ER in the presence of different β1-subunits was analyzed (Fig. 3, *B* and *C*). Expression of ZERO alone resulted in a Pearson's correlation coefficient of 0.82 ± 0.03 (*n* = 8) for ZERO with ER. This was significantly reduced upon co-expression of wild-type β1 to 0.53 ± 0.05 (*n* = 8), revealing that β1-subunits promote ER exit of ZERO. The ER colocalization of ZERO in the presence of the N-terminal truncated β1-subunit (β1-Δ17-myc_c_) was not significantly different from ER localization of ZERO in the absence of β1-subunits (ER colocalization was 0.82 ± 0.02; *n* = 8) (Fig. 3, *B* and *C*). Co-expression of ZERO with the β1-L17Α subunit resulted in an ER colocalization of ZERO that was intermediate between ER colocalization of ZERO expressed alone and in the presence of wild-type β1-subunits at 0.65 ± 0.05 (*n* = 8; Fig. 3, *B* and *C*). This partial effect of the L17A mutation on ER trapping is in agreement with the incomplete (∼50%) suppression of β1-subunit enhanced BK channel α-subunit surface expression conferred by the L17A mutation compared with complete deletion of the N terminus (compare β1-Δ17-myc_c_ in Fig. 1*C* with β1-L17Α in Fig. 3*A*). Thus, although Leu-17 plays an important role in controlling BK channel surface expression and contributes to ER exit of the ZERO α-subunit, additional features conferred by the most membrane-proximal N-terminal residues (*i.e.* after residue 14) are also required for efficient ER exit and surface trafficking.

To examine whether the reduction in ER export in the presence of the β1-subunit N terminus or the L17A mutant is a result of a defect of ER exit of the β1-subunit itself, we performed cell surface expression and ER colocalization using the β1-subunit mutant with an additional extracellular myc tag as above (Fig. 2*B*). Compared with wild-type β1-subunit alone, surface expression of the β1-Δ17-myc_e&c_ and the β1-L17A-myc_e&c_ was only 10.3 ± 1.2 (*n* = 4) and 48.4 ± 6.7% (*n* = 4), respectively, of wild-type β1-subunit surface expression (Fig. 2*B*). In addition, co-expression of either β1-Δ17-myc_c_ orβ1-L17Α-myc_c_ alone with the ER expression vector pDsRed-ER increased ER colocalization of the mutant β1-subunit when compared with the wild-type β1-subunit. The *R* value of wild-type β1 was 0.66 ± 0.05 (*n* = 4), whereas the values for mutant β1-Δ17-myc_e&c_ and β1-L17Α-myc_e&c_ were 0.85 ± 0.02 (*n* = 4) and 0.82 ± 0.02 (*n* = 4), respectively. Thus, at least in part, the reduced cell surface expression of ZERO subunits promoted by β1-subunits is likely dependent upon the ability of the β1-subunit to exit the ER itself.

### Membrane-distal residues of the β1-subunit N terminus determine functional coupling with α-subunits

**Figure 4.**
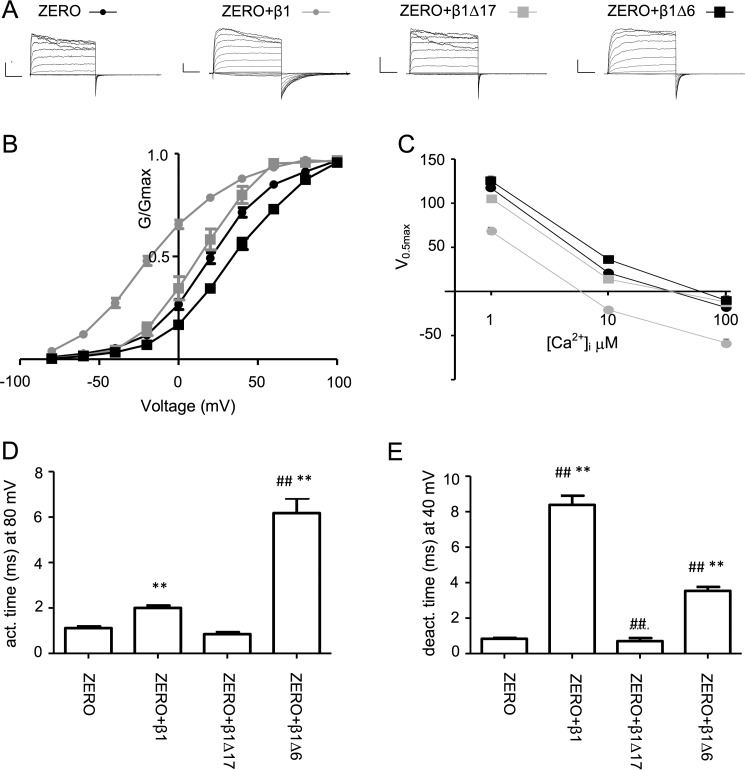
**First six residues of β1-subunit N terminus are essential for functional coupling with α-subunit ZERO variant.** *A*, representative macropatch current recordings from excised inside-out patches from HEK293 cells expressing the ZERO variant and respective β1-subunit mutants in the presence of 10 μm intracellular free calcium with voltage protocols as described under “Experimental procedures.” *B*, *G*/*V* relationship for constructs as in *A* with curve fits using the Boltzmann equation (see “Experimental procedures”). *C*, voltage of half-maximal conductance (*V*_0.5max_) in the presence of 1, 10, or 100 μm intracellular free calcium. *D* and *E*, activation (*act.*) time constant (*D*) and deactivation (*deact.*) time constant (*E*) for the respective ZERO and β1-subunit combinations in *A*. Data are means, and *error bars* represent S.E. (*N* > 10/group). **, *p* < 0.01 compared with ZERO alone; ##, *p* < 0.01 compared with ZERO + wild-type β1-myc_c_ (ANOVA with Dunnett's post hoc test).

In accordance with previous data revealing the importance of an intact N terminus of the β1-subunits for functional coupling to α-subunits (13, 14), deletion of the entire N terminus (β1-Δ17) abolished both the significant left shift of the conductance voltage curve and slowing of activation and deactivation observed across all intracellular free Ca^2+^ concentrations analyzed ranging from 1 to 100 μm (Fig. 4).

In the presence of 10 μm intracellular free Ca^2+^, the *V*_0.5max_ of ZERO expressed alone was 19.8 ± 1.2 mV (*n* = 33; Fig. 4, *B* and *C*). This was left shifted by ∼40 mV to −20.5 ± 1.5 mV (*n* = 45) by the wild-type β1-myc_c_ subunit but not by the mutant β1-Δ17-myc_c_ (*V*_0.5max_ was 12.2 ± 1.9 mV; *n* = 14) (Fig. 4, *B* and *C*). In control experiments, we observed that the presence of the -myc_c_ tag attenuated the left shift induced by β1-subunits by 17.7 mV. The activation time constants (determined at 80 mV) and deactivation time constants (determined at 40 mV) of ZERO (*n* = 33) were both significantly slowed by wild-type β1-myc_c_ (*n* = 45) subunit from 1.12 ± 0.07 and 0.84 ± 0.06 ms to 2.00 ± 0.11 and 8.39 ± 0.52 ms, respectively (Fig. 4, *D* and *E*). However, both activation and deactivation time constants (0.85 ± 0.09 and 0.70 ± 0.18 ms, respectively) were not significantly shifted in the presence of β1-Δ17-myc_c_ (*n* = 14; Fig. 4, *D* and *E*). These results confirm that the intracellular N terminus of β1 is essential for its regulation of the electrophysiological properties of BK channels.

To examine whether deletion of the first six residues of the N terminus, which do not affect β1-subunit-mediated enhancement of ZERO trafficking, would change its functional coupling with α-subunits, we co-expressed the mutant β1-Δ6-myc_c_ subunit with the ZERO variant. In contrast to the left shift mediated by wild-type β1-subunits, β1-Δ6-myc_c_ induced a small but significant right shift in *V*_0.5max_ to 35.7 ± 1.6 mV (*n* = 14) when compared with ZERO alone (Fig. 4, *B* and *C*). Therefore, the absence of the first six residues of β1-subunit completely abolished the left shift of the conductance-voltage (*G*/*V*) relationship induced by the wild-type β1. These data reveal that distinct domains of the N terminus control functional coupling and trafficking regulated by β1-subunits. However, although β1-Δ6-myc_c_ still significantly slowed BK channel activation and deactivation compared with ZERO alone, BK channel activation and deactivation rates in the presence of β1-Δ6-myc_c_ were significantly different compared with those in the presence wild-type β1-myc_c_ (Fig. 4, *D* and *E*). BK channel activation was slowed to a greater extent with β1-Δ6-myc_c_ compared with wild-type β1-myc_c_, whereas deactivation was faster with β1-Δ6-myc_c_ compared with wild-type β1-myc_c_.

### Does the N terminus of the β1-subunit form an amphipathic α-helix to control functional coupling?

**Figure 5.**
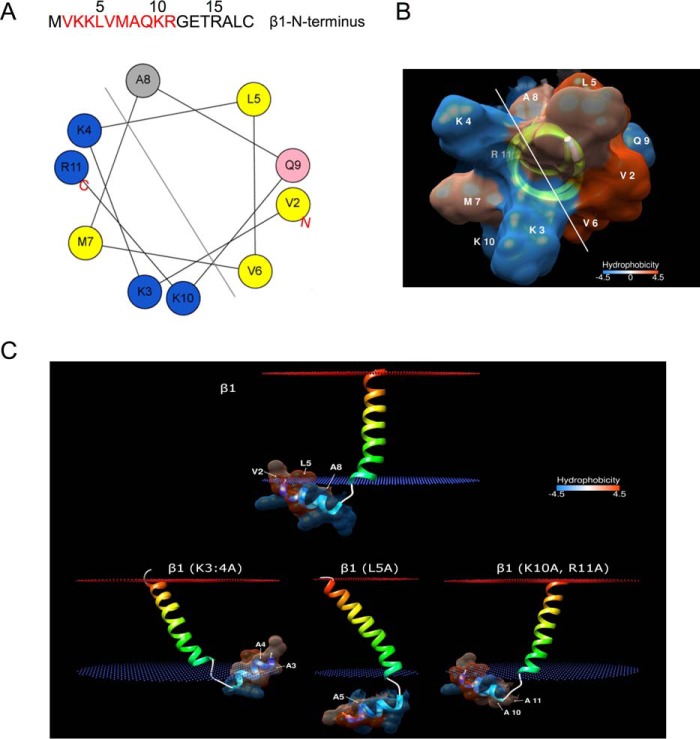
**Does the β1-subunit N terminus form an amphipathic helix?** *A*, helical wheel depiction of the N-terminal amphipathic α-helix (residues 2–11). The color code for residues is as follows: *yellow*, hydrophobic; *purple*, serine and threonine; *blue*, basic; *red*, acidic; *pink*, asparagine and glutamine; *gray*, alanine and glycine; *green*, proline; *light blue*, histidine. *B*, hydrophobicity of the amphipathic α-helix represented by a *red* and *blue* scale, respectively. *C*, molecular system of the β1-subunit N terminus and TM1 interaction with a membrane lipid bilayer (*blue* and *red dotted* outer and inner membrane, respectively). The hydrophobic Val-2, Leu-5, and Ala-8 on the amphipathic N-terminal helix are anchored onto the membranes. Increased interaction between the N terminus and membrane occurs when charged polar residues are mutated to hydrophobic residues. Leu-5 is predicted to be vital for this interaction.

The first six residues of the β1-subunit include a pair of basic residues (Lys-3 and Lys-4) that are important for gating in the absence of calcium (10), most likely dependent on the interaction of these residues with negative charges. In an attempt to further understand why the first six residues of the N terminus are important for functional coupling, we analyzed the computationally modeled secondary and tertiary structure of the N terminus and the first transmembrane domain of the β1-subunit embedded within the lipid bilayers of an artificial membrane. Increasing evidence suggests that regions of the β1 N terminus are likely to interact with the α-subunit (*e.g.* see Refs. 15 and 16) and that the orientation of the β1-subunit transmembrane domains is likely to be important for interaction and coupling. Residues 2–11 of the β1-subunit N terminus display the characteristic spatial segregation of hydrophilic and hydrophobic amino acids of an amphipathic α-helix (Fig. 5*A*). Furthermore, the tertiary structure of the N terminus of β1-subunit, connected through an extended flexible loop with its first transmembrane domain, suggests that the extreme end of the intracellular N-terminal region is likely to fold into three helical turns with opposing hydrophobic and hydrophilic surfaces (Fig. 5*B*).

When a molecular system consisting of membrane·β1-subunit complex was simulated, the hydrophilic surface formed by the polar residues Lys-3, Lys-4, Lys-10, and Arg-11 of β1-subunit is predicted to be oriented away from the membrane facing the cytoplasm, whereas the hydrophobic surface of residues Val-2, Leu-5, and Ala-8 serves as a potential membrane anchorage site that supports the association of the intracellular N terminus with the membrane (Fig. 5*C*). The anchorage is predicted to occur when residues Val-2, Leu-5, and Ala-8 insert themselves into the membrane where their side chains may interact with those of the membrane lipid bilayers. This hydrophobic interaction was markedly affected when the interacting residues were mutated in our modeled molecular systems. For instance, the anchorage was severely disrupted when Leu-5 was mutated to a far less hydrophobic neutral alanine (Fig. 5*C*), whereas a similar change in V6A (not shown) and K10A,R11A on the cytoplasm-facing hydrophilic surface did not have any significant impact on the hydrophobic interaction between the helix and membrane (Fig. 5*C*).

However, the helical structure of the K3A,K4A mutant was rotated, and together with other hydrophobic residues they form a much stronger hydrophobic interaction with the membrane. If such a membrane interface (or potential interface with regions of the α-subunit) do indeed exist in the channel complex, the interaction between the amphipathic α-helical N terminus and the membrane may therefore offer a mechanistic insight into the underlying functional coupling between α-subunits and β1-subunits. Importantly, such a model would predict that mutation of the basic Lys-3 and Lys-4 (as well as hydrophobic Leu-5) but not Lys-10 and Arg-11 residues would control functional coupling and support the functional data above that the first six residues of the N terminus are involved in functional coupling and distinct from those controlling β1-subunit -mediated enhancement of α-subunit cell surface expression.

**Figure 6.**
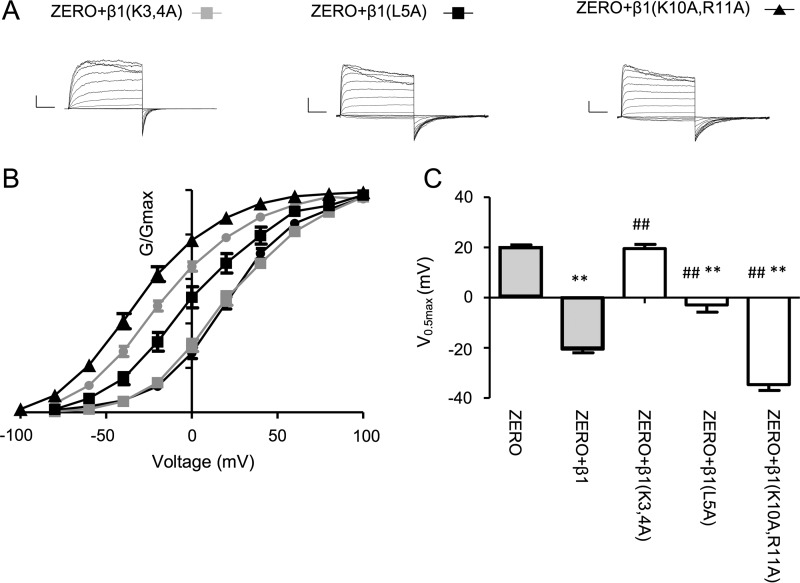
**Lys-3, Lys-4, and Leu-5 of β1-subunit N terminus are important determinants of functional coupling.** *A*, representative macropatch current recordings from excised inside-out patches from HEK293 cells expressing the ZERO variant and respective β1-subunit mutants in the presence of 10 μm intracellular free calcium with voltage protocols as described under “Experimental procedures.” *B* and *C*, *G*/*V* relationship for constructs with curve fits using the Boltzmann equation (see “Experimental procedures”) (*B*) and voltage of half-maximal conductance (*C*) for the respective ZERO and β1-subunit combinations in *A*. The data for ZERO (*shaded box*) and ZERO with wild-type β1-subunit (*shaded box*) are from Fig. 4 and shown for comparison. Data are means, and *error bars* represent S.E. (*N* > 10/group). **, *p* < 0.01 compared with ZERO alone; ##, *p* < 0.01 compared with ZERO + wild-type β1-myc_c_ (ANOVA with Dunnett's post hoc test).

To test this model, we examined whether mutating Lys-3 and Lys-4 to alanine (β1-(K3A,K4A)-myc_c_) would result in the loss of functional coupling as predicted and observed in the β1-Δ6-myc_c_ deletion mutant. In the presence of β1-(K3A,K4A)-myc_c_, there was no significant left shift of the *G*/*V* relationship of ZERO, resulting in a *V*_0.5max_ of 19.6 ± 1.6 mV (*n* = 11), similar to ZERO alone under these conditions (Fig. 6, *A–C*). In further support of the model, mutation of the more membrane-proximal basic residues to alanine (K10A,R11A) still significantly left-shifted the *G*/*V* relationship of ZERO to a *V*_0.5max_ of −31.2 ± 5.1 mV (*n* = 10), a value in fact slightly more left-shifted compared with wild-type β1-myc_c_ (Fig. 6, *B* and *C*). Thus, as predicted by the modeling, the lack of functional regulation by β1-(K3A,K4A)-myc_c_ was specific to these residues rather than a general effect of changing overall charge of the N terminus.

We also examined the structural impact of mutating the patch of hydrophobic residues immediately downstream of Lys-3 and Lys-4, leucine (Leu-5), and valine (Val-6), which have also been implicated in functional coupling (12). As described above, the modeling (Fig. 5*C*) predicted a conformational rearrangement of the β1-subunit upon mutation of Leu-5 to alanine (β1-L5A-myc_c_) but not Val-6 to alanine (β1-V6A-myc_c_). Indeed, co-expression of the β1-L5A-myc_c_ subunit resulted in a significantly attenuated left shift in *V*_0.5max_ to only −2.9 ± 2.7 mV (*n* = 10). This shift was intermediate between that of ZERO with β1-myc_c_ and ZERO alone (Fig. 6, *A*, *B*, and *C*), indicating that the L5A mutation significantly inhibited, but did not totally abolish, the effects found with wild-type β1-subunit on BK channels on *V*_0.5max._ Moreover, with the β1-L5A-myc_c_ subunit, BK channel activation and deactivation were still significantly slowed to 1.8 ± 0.1 and 5.9 ± 0.9 ms, respectively (*n* = 10). These values were similar to the effect of wild-type β1 (see Fig. 4, *D* and *E*). Mutation of the only other leucine residue in the N terminus, Leu-17, which we identified in our cell surface expression assays to be important for β1-subunit-mediated trafficking of ZERO, to alanine had no effect on functional coupling. β1-L17A-myc_c_ behaved similarly to wild-type β1-myc_c_ subunit by left-shifting the *G*/*V* relationship to a *V*_0.5max_ of −26.3 ± 3.1 mV (*n* = 10). Again, as predicted by the structural modeling, mutation of Val-6 to alanine (β1-V6A-myc_c_) also resulted in a significant left shift in *V*_0.5max_ (to −29.7 ± 5.6 mV, *n* = 10), comparable with that observed with wild-type β1-myc_c._ Taken together, these data reveal that distinct residues on the β1-subunit N terminus control trafficking (membrane-proximal Leu-17) and functional coupling (membrane-distal Lys-3, Lys-4, and Leu-5), respectively.

## Discussion

These data reveal that different residues on the short intracellular N terminus of β1-subunits control trafficking and functional regulation of BK channel α-subunits. The membrane-proximal amino acids of the β1-subunit N terminus are important for the enhancement of BK α-subunit surface expression with Leu-17 playing an important role and residues distal to residue 14 being dispensable for this trafficking effect. In contrast, membrane-distal residues play a critical role in functional coupling with the basic lysine pair (Lys-3 and Lys-4) as well as the downstream hydrophobic leucine (Leu-5) having an important role.

β1-subunits have been reported to exert diverse effects on BK channel α-subunit cell surface expression with both inhibition and enhancement of α-subunit surface expression reported. For example, an endocytic motif in the β1-subunit C terminus has been reported to suppress cell surface expression of hSlo α-subunits, whereas the avian β1-subunit enhances surface expression of the α-subunit (7, 8). This divergence of mechanism is likely a function of the α-subunit splice variant. β1-subunits enhanced the surface expression of α-subunit splice variants with a C-terminal -DEC motif (7), whereas suppression of surface expression was undertaken using the QEERL C-terminal α-subunit variant (8). Moreover, assembly of β1-subunits with α-subunits appears to be dynamic including via signaling-dependent control of β1-subunit trafficking from rab11a-positive endosomes as well as assembly in the endoplasmic reticulum (24). In our system, using the ZERO α-subunit splice variant that includes the -DEC C-terminal motif, mammalian β1-subunit enhanced the surface expression of the ZERO variant isoform more than 2-fold, a mechanism that is dependent on an intact N terminus of the β1-subunit that, at least in part, determines the efficiency of α-subunit exit from the ER.

Importantly, the first 14 residues of the N terminus were dispensable for this enhancement of surface trafficking of α-subunits with leucine 17 being identified as the only critical residue in the most membrane-proximal residues of the N terminus. In the avian system in which the β1-subunit has a predicted longer N terminus and only ∼40% sequence conservation, the first 42 amino acids (encompassing the N terminus and TM1) were sufficient to promote surface trafficking of α-subunits (7). Intriguingly, a membrane-proximal leucine residue (at position 20 in avian β1 and the corresponding Leu-17 in murine β1) was also an important determinant of cell surface expression, suggesting a potential common mechanism (7). In a number of different proteins, N-terminal leucine residues can contribute to atypical dileucine-like sorting motifs (25). Although mutation of residues upstream of the important Leu-17 to alanine had no significant effect on the enhancement of α-subunit trafficking, whether Leu-17 is part of a trafficking motif or may play a more structural role, for example in controlling TM1 orientation in the plasma membrane, which may be important for ER exit as suggested for β4-subunits (26), remains to be determined. Moreover, the L17A mutant, in contrast to deletion of the entire β1 N terminus, had only a partial effect on α-subunit (or β1-subunit itself) surface expression. Thus Leu-17 may also play an important role in post-ER mechanisms of α-subunit/β1-subunit sorting or assembly (24).

In contrast to the role of Leu-17 in trafficking, our data revealed that Leu-17 is not important for functional coupling. Although it has long been appreciated that the cytosolic N terminus of the β1-subunit is required for functional coupling with α-subunits (*e.g.* see Refs. 13 and 14), only relatively recently have specific residues in the β1-subunit N terminus been defined as important contributors to functional coupling. For example, the basic residues Lys-3 and Lys-4 have been proposed to provide a specific mechanism to stabilize the voltage sensor of α-subunits such that when mutated to alanine gating charge movements were abolished (10) without affecting the conductance-voltage relationship in the absence of calcium. These data suggested that Lys-3 and Lys-4 interact with a cytosolic domain of the α-subunit. Moreover, Liu *et al.* (12) reported that both the adjacent hydrophobic motif of Leu-5, Val-6, and Met-7 and an electrostatic site formed by Glu-13 and Thr-14 interacted with the C terminus of α-subunit to control calcium sensitivity of BK channels. Indeed, independent biophysical and biochemical approaches suggest that the cytosolic domains of β1-subunits must be close to the voltage-sensing domains of α-subunits (15, 16, 27). Furthermore, these data support that the transmembrane segments of β1-subunits (TM1 and TM2) are spatially arranged close to the S0 transmembrane domain of α-subunits. Taken together, the membrane-distal residues of the β1-subunit N terminus are likely to act as the key determinants of functional coupling of β1-subunits with α-subunits. As both TM1 and TM2 of β1-subunits are required for functional coupling (28), this would further suggest that the correct spatial organization of these transmembrane domains with the α-subunit is required for functional regulation.

Importantly, the predicted amphipathic helix on the N-terminal membrane-proximal domain of β1-subunit may provide the structural basis for the important roles the basic Lys-3 and Lys-4 residues and the hydrophobic patch play in perhaps controlling the correct spatial orientation of the β1-subunit within the α-subunit complex. Whether this involves interaction with multiple domains of the α-subunit or providing a β1-subunit-dependent interface between the plasma membrane and α-subunit remains to be determined.

Intriguingly, the specific residues involved in β1-subunit-dependent trafficking and functional coupling with α-subunits are also largely distinct from N-terminal residues of β1-subunit that are involved in regulation by other molecules such as ω-3 fatty acids (17). However, in the case of ω-3 fatty acids, potential interactions between Arg-15 and Cys-18 in the most membrane-proximal domain of the N terminus are important, suggesting that functional integrity of the same membrane-proximal domain required for efficient trafficking is important. Interestingly, recent reports have indicated somatic mutations in cancer alter both Lys-4 and Leu-5 residues (19, 20), and changes in BK channel activity have been linked to cancer cell proliferation and migration (21–23). However, whether mutation of Lys-4 and/or Leu-5 is involved in regulating native BK channel activity, cancer growth, or migration in tissues expressing β1-subunits is not known.

Clearly, investigation of whether mutations of N-terminal β1-subunits residues are observed in other diseases or of signaling molecules that differentially regulate BK channel function by targeting domains of the β1 will be of interest. Finally, convergence of multiple mechanisms to control BK channel properties, function, and regulation on the short intracellular N terminus of β1-subunits further supports the role of β1-subunits in specifying cell-specific function and behaviors.

## Experimental procedures

### Expression constructs

Plasmids encoding the full-length BK channel α-subunit ZERO splice variant with either an N-terminal FLAG tag or both a FLAG- and a C-terminal HA (-HA) tag were described previously (26).

A sticky-end PCR cloning strategy was adopted to generate -myc_c_-tagged β1-subunit constructs in the pcDNA3.1 vector. The human β1-subunit was first amplified from a construct (a generous gift from Dr. Jon Lippiat, University of Leeds; Ref. 29) using primers NheI-β1 fwd (5′-ACTCAGATCTGCTAGCATGGTGAAGAAG) and β1-myc-XhoI rev (5′-ACTCGAGCTAAGATCCTCTTCTGAGATGAGTTTTTGTTCCTTCTGGGCCG). To make a construct with both extracellular and C-terminal *myc* tags (myc_c&e_), site-directed mutagenesis with primers 5′-AGCCAAATTCCAAGAGCAGAAACTCATCTCAGAAGAGGATCTTCAGG (fwd) and 5′-AAGCAGTAGAAGACCTGAAGATCCTCTTCTGAGATGAGTTTCTGC (rev) were used to introduce this additional myc_e_ tag between Gln-131 and Gln-132 of β1-subunit. These two constructs then served as the templates to generate additional constructs with mutated intracellular N termini using either the KOD mutagenesis kit (Novagen) or PCR subcloning of amplicons with NheI and XhoI restriction tails. Primers used for the mutagenesis were as follows: β1-Δ6: fwd, 5′-AAGCTGGCTAGCCACGGTGAAGAAGCTG, and BGH rev (Life Technologies);β1-Δ17: fwd, 5′-AGCTGGCTAGCCAACATGCTGGGTGTAACCATGG, and BGH rev; β1-(K3,4A): fwd, 5′-AGATCTACATGGTGGCGGCGCTGGTGATGGCCCAGAAG, and rev, 5′-TCTGGGCCATCACCAGCGCCGCCACCATGTAGATCTGAG; β1-L5A: fwd, 5′-TACATGGTGAAGAAGGCGGTGATGGCCCAGAAG, and rev, 5′-TCTGGGCCATCACCGCCTTCTTCACCATGTAG; β1-V6A: fwd, 5′-ATGGTGAAGAAGCTGGCGATGGCCCAGAAGCG, and rev, 5′-GCTTCTGGGCCATCGCCAGCTTCTTCACCATG; β1-(K10A,R11A): fwd, 5′-TGGTGATGGCCCAGGCAGCAGGAGAGACACGAG, and rev, 5′-TCGTGTCTCTCCTGCTGCCTGGGCCATCACCAG; β1-(R11A,G12A,E13A): fwd, 5′-TGGTGATGGCCCAGAAGGCGGCAGCGACACGAGCCCTTTGCC, and rev, 5′-AGGCAAAGGGCTCGTGTCGCTGCCGCCTTCTGGGCCATCACC; β1-T14A: fwd, 5′-AGAAGCGGGGAGAGACACGAGCCCTTTGCC, and rev, 5′-AGGCAAAGGGCTCGTGCCTCTCCCCGCTTC; β1-R15A: fwd, 5′-AGAAGCGGGGAGAGACAGCAGCCCTTTGCCTG, and rev, 5′-AGGCAAAGGGCTGCTGTCTCTCCCCGCTTCTG; β1-L17A: fwd, 5′-AGAGACACGAGCCGCTTGCCTGGGTGTAACC, and rev, 5′-TACACCCAGGCAAGCGGCTCGTGTCTCTCC; and β1-C18A: fwd, 5′-AGAGACACGAGCCCTTGCCCTGGGTGTAACCATG, and rev, 5′-ATGGTTACACCCAGGGCAAGGGCTCGTGTCTCTC-3′. All constructs were verified by sequencing.

### Cell culture, transfection, and imaging

HEK293 cells were cultured and transfected with Lipofectamine 2000 according to a previously published protocol (26). Quantitative cell surface labeling of N-terminal FLAG epitope-tagged BK channel α-subunits in non-permeabilized cells was performed using mouse monoclonal anti-FLAG M2 antibody (Sigma) and secondary anti-mouse Alexa Fluor 543 (Invitrogen) at a dilution ratio of 1:100 and 1:1000, respectively. Cells were then fixed in 4% paraformaldehyde for 30 min, permeabilized with 3% Triton X-100 for 10 min, and blocked with phosphate-buffered saline containing 3% bovine serum albumin plus 0.05% Tween 20 for 1 h. For total BK channel expression, either the intracellular C-terminal HA epitope tag was probed with anti-HA polyclonal rabbit antibody (Zymed Laboratories Inc.; 1:500) followed by Alexa Fluor 647 (Molecular Probes; 1:1000), or the FLAG tag was probed with anti-FLAG antibody with anti-mouse Alexa Fluor 488 (1:1000).

Quantitative membrane expression of β1-subunits with both myc_c_ and myc_e_ tags was detected using rabbit anti-myc (Immune Systems) at 1:300 and anti-rabbit secondary antibody conjugated to Alexa Fluor 546 prior to fixation and permeabilization. Total expression of β1-subunits was probed with rabbit anti-myc antibody (Immune Systems) at 1:1000 and anti-rabbit secondary antibody conjugated to Alexa Fluor 488 at 1:1000. Co-expression of α- and β1-subunits was determined as above with the exception that the anti-rabbit secondary antibody was conjugated to Alexa Fluor 647. Cells were mounted in Mowiol and dried at room temperature overnight in the dark before image acquisition.

Confocal images were acquired on a Zeiss LSM510 laser-scanning microscope using a 63 × oil Plan Apochromat (numerical aperture, 1.4) objective lens at Nyquist sampling rates in multitracking mode to minimize channel cross-talk. 3D image stacks were deconvolved using the program Huygens (Scientific Volume Imaging), and cell surface expression of full-length channels was determined by quantitative immunofluorescence calculated as the surface (FLAG-) to total channel protein (-HA or intracellular FLAG-) ratio using ImageJ (National Institutes of Health). For each cell, the absolute mean FLAG- and -HA immunofluorescence signal was determined following background subtraction in each channel. The absolute FLAG- to -HA ratio was then calculated and normalized to the ratio determined for the respective control as indicated in each figure that was run in parallel in each experiment. In each independent transfection (*N*), multiple cells were randomly selected, and an experiment average was determined for each construct. For statistical analysis, the average ratio in each independent experiment was used to calculate each group mean.

Colocalization of the different mutant β1-subunits with the ER was assayed by co-transfecting the subunits with pDsRed-ER expressing the ER marker (Clontech). Confocal images were acquired and deconvolved as above, and Pearson's correlation coefficient (*R*) was determined using ImageJ with an *R* value of +1 indicating 100% colocalization.

### Electrophysiology

Macropatch recordings were performed using the inside-out patch clamp configuration at room temperature essentially as described previously (30). Briefly, the extracellular recording solution was composed of 140 mm KMeSO_3_, 2 mm KCl, 20 mm HEPES, 2 mm MgCl_2_, pH 7.3. Internal solution was composed of 140 mm KMeSO3, 2 mm KCl, 20 mm HEPES, 5 mm HEDTA, pH 7.3, with CaCl_2_ added to give a free Ca^2+^ concentration of 10 μm. Voltage protocols and acquisition were controlled using an Axopatch 200B amplifier and Digidata 1440 using pCLAMP10. *G*/*V* relationships were constructed from tail currents recorded using a holding potential of −120 mV with 100-ms steps over a voltage range of −100 to +120 mV in 20-mV steps followed by a step back to −80 mV. *V*_0.5max_ was determined from Boltzmann fits of the normalized *G*/*V* curves using the following equation.

$$G/G_{\max}=1/\left(1+\exp\left(-ze\left(V-V_{0.5\;\max}\right)/kT\right)\right)$$

where *z* is the number of equivalent gating charges, *V*_0.5max_ is the voltage for half-activation of the channel, *e* is the elementary charge, *k* is the Boltzmann constant, and *T* is the absolute temperature. Activation and deactivation time constants were determined by fitting activation and deactivation curves to an exponential function.

### Statistical analysis

All data are presented as means ± S.E. with *N* representing the number of independent imaging experiments or cells in electrophysiology assays and *n* representing the number of individual cells analyzed in imaging assays. Data from independent cells in electrophysiology experiments or independent experiments in imaging assays (*N*) were analyzed by ANOVA with post hoc Dunnett's test having significance set as follows: *, *p* < 0.05; **, *p* < 0.01; and ***, *p* < 0.001.

### In silico structural analysis

Secondary structure of the wild-type β1-subunit was predicted by submitting its protein sequence to the JPred online tool (31). Amphipathicity and potential helical secondary structure on the N-terminal region was analyzed with Heliquest (32). The initial tertiary structure of the first 50 N-terminal residues of the wild-type β1-subunit was modeled using the I-Tasser web server (33). A number of probable models were built by the server based on multiple threading alignments followed by iterative assemblies of template fragments. The structure with the highest confidence score was next chosen to be further refined using the Swiss-PdbViewer Version 4.1 (34). Briefly, the last 10 residues of the modeled peptide were first trimmed to better reflect the predicted and reported consensus secondary structure before several rounds of side-chain fixations and energy minimization were applied sequentially until no further improvement could be achieved. Quality of the enhanced structure was assessed on both the WHAT-IF (35) and SWISS-MODEL structural assessment (36) web servers. This predicted wild-type model was then used as a template in the homology modeling of the mutated N-terminal β1 peptides using the Multiple Mapping Method (MMM) web server (37). MMM combines the best alternatively aligned fragments along the peptide into a final alignment used for the model construction. All the predicted mutant models were further refined and assessed as above. The rotational and translational orientation of each of the modeled peptide with respect to membrane lipid bilayers was next calculated using the PPM web server (38). The peptide/membrane molecular system was subsequently assembled with the lipid replacement method, equilibrated, and minimal dynamics-simulated in a synthetic environment of 1-palmitoyl-2-oleoyl-*sn*-glycero-3-phosphocholine lipid bilayers using the CHARM-GUI web server (39). The 3D models were visualized using the program Chimera (40).

## Author contributions

M. J. S. conceived and designed the study with L. C. M. J. S. coordinated and, with L. C., wrote the manuscript. L. C. generated constructs and performed and analyzed imaging and biochemical assays. D. B. performed and analyzed electrophysiological experiments. Z. H. L. performed the *in silico* modeling and provided the bioinformatics discussion. H. M. generated constructs and analyzed and produced data in Fig. 1. All authors reviewed the results, edited drafts, and approved the final version of the manuscript.

## Footnotes

2 The abbreviations used are:

BK: large-conductance calcium- and voltage-activated potassium
TM: transmembrane-spanning domain
ER: endoplasmic reticulum
*G*/*V*: conductance-voltage
myc_c_: C-terminal myc
myc_e_: extracellular myc
fwd: forward
rev: reverse
ANOVA: analysis of variance
BGH: bovine growth hormone.
